# Three tandem promoters, together with IHF, regulate growth phase dependent expression of the *Escherichia coli kps* capsule gene cluster

**DOI:** 10.1038/s41598-017-17891-0

**Published:** 2017-12-20

**Authors:** Jia Jia, Jane E. King, Marie C. Goldrick, Esraa Aldawood, Ian S. Roberts

**Affiliations:** 1 School of Biological Sciences, Faculty of Biology Medicine and Health, Manchester Academic Health Science Centre, University of Manchester, Manchester, M13 9PT UK; 2 Nanjing Medical University, School, 140 Hanzhong Road, Nanjing, 210029 China

## Abstract

In this study we characterise three tandem promoters (PR1-1, PR1-2 and PR1-3) within the PR1 regulatory region of the *Escherichia coli kps* capsule gene cluster. Transcription from promoter PR1-2 was dependent on the activity of the upstream promoter PR1-1, which activated PR1-2 via transcription coupled DNA supercoiling. During growth at 37 °C a temporal pattern of transcription from all three promoters was observed with maximum transcriptional activity evident during mid-exponential phase followed by a sharp decrease in activity as the cells enter stationary phase. The growth phase dependent transcription was regulated by Integration Host Factor (IHF), which bound within the PR1 region to repress transcription from PR1-2 and PR1-3. This pattern of transcription was mirrored by growth phase dependent expression of the K1 capsule. Overall these data reveal a complex pattern of transcriptional regulation for an important virulence factor with IHF playing a role in regulating growth phase expression.

## Introduction

*Escherichia coli* isolates from extraintestinal infections express a polysaccharide capsule or K antigen^1^. Capsule expression is an important virulence factor providing a selective advantage to the bacteria. It has been implicated in aiding transmission between hosts by preventing desiccation^2–4^ in adhesion^5^, resistance to host’s innate defences^6–9^, resistance to the host’s adaptive immune response^10–12^, and for intracellular survival, and crossing the blood brain barrier^13,14^. In addition, polysaccharide capsules also play signaling roles in mediating interactions between the pathogen and the host^15^. These include moderating induction of chemokines and cytokines, interacting with toll-like receptors (TLRs), and the perturbation of mucus clearance^16^.

**Figure 1:**
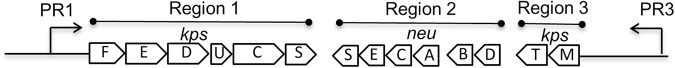
Transcriptional and genetic organisation of the K1 capsule gene cluster. The gene cluster is composed of three regions. Region 1 (*kps FEDUCS*) and Region 3 (*kps MT*) are conserved throughout Group 2 capsules while Region 2 is serotype specific. The genes are transcribed from two major convergent promoters PR1 and PR3.

*E*. *coli* capsules have been classified into four groups (Groups 1–4) on the basis of a number of biochemical and genetic criteria^17^. The Group 2 capsule gene cluster (*kps*) is composed of three regions (Fig. 1) in which a serotype-specific region 2 is flanked by two conserved regions: region 1 and region 3 that contain the genes responsible for transport of newly synthesized capsular polysaccharides from the cytoplasm to the bacterial cell surface^18,19^. Region 2 encodes the genes responsible for the synthesis of the specific capsular polysaccharide and its precursors^18^. Expression of group 2 capsule gene clusters is driven by two temperature-regulated promoter regions, Promoter Region 1 (PR1), located 225 bp upstream of *kpsF*, and Promoter Region 3 (PR3), 714 bp upstream of *kpsM* (Fig. 1)^20–22^. Regulation of expression is complex involving H-NS, IHF, RfaH and SlyA^21–23^. In both PR1 and PR3 there is a long untranslated region (UTR) that, in both cases, appears to be important in temperature regulation^22,23^ and in the case of PR3 important in attenuating the level of transcription that reaches *kpsM* ^22^. In the case of PR1 two putative additional transcriptional start points have been located within the 225 bp UTR^20,24^. However whether these represent functional promoters and what role they may play in the regulation of transcription of region 1 is not known.

In this paper we present a detailed analysis of the PR1 region. We demonstrate the presence of three functional promoters PR1-1, PR1-2 and PR1-3 that contribute to *kps* expression and show that maximum PR1-2 activity is dependent on the transcription from the upstream PR1-1 probably through transcription-coupled supercoiling, while promoter PR1-3 can act independently. We show the contribution of each promoter to the temporal pattern of transcription during bacterial growth at 37 °C with a burst of transcription occurring in mid exponential phase which decreases as the cells enter stationary phase, a pattern mirrored by the cell surface expression of capsular polysaccharide. IHF binding in PR1 is critical to the growth phase regulation of transcription acting to repress transcription from PR1-2 and PR1-3 at the end of exponential phase. Taken as a whole these results add a new level of complexity to our understanding of regulation of transcription from the PR1 region, its regulation, and its role in the growth phase regulation of this capsule expression in pathogenic *E*. *coli*.

## Results

### Characterisation of promoters within in the 5′ UTR at PR1 and growth phase dependent transcription

**Figure 2:**
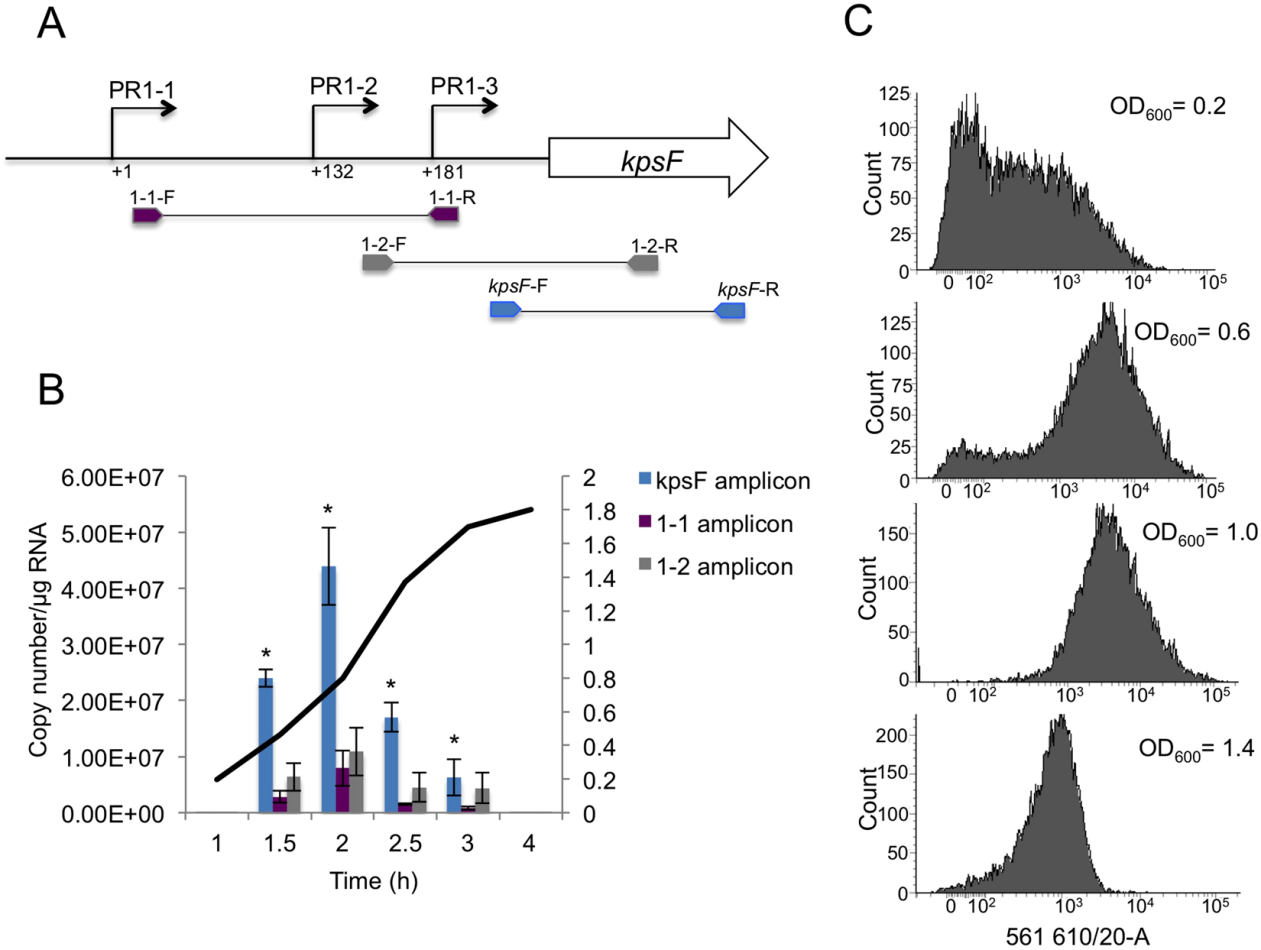
Quantification of transcripts from PR1-1, PR1-2 and PR1-3 during the different growth phases of UTI89. (**A**) Illustration of the qRT-PCR primers used and their corresponding amplicons. (**B**) Growth curve of UTI89 and at the time points indicated RNA was extracted and qRT-PCR was performed using the primer sets shown. The *kpsF* amplicon represents the total transcript coming from all three promoters; the 1-1 amplicon represents transcription from just PR1-1 while the 1-2 amplicon represents transcription from both PR1-1 and PR1-2. Values are the mean of four independent experiments (normalised against *rpoD* and 16 s transcripts) and error bars represent the standard error of the mean. For simplicity the growth curve is a representative of one independent experiment but OD_600_ values were within 0.05 units for each quadruplicate sample. The asterisks denote significance (*p* < 0.05) between the copy number of the Kps amplicon at each time point. (**C**) Flow cytometry analyses performed on UTI89 at the indicated OD_600_ using anti-K1 antibody and Alexa Fluor 594 labelled secondary antibody. An increase in fluorescence intensity and hence surface capsule can clearly be seen as the cells grow but this intensity decreases towards the end of exponential growth. There was no statistically significant change (*p* > 0.05) in the length of strain UTI89 at each of the four time points examined by FACS, with the average length at each time point being 0.95 ± 0.1 μm.

Previous studies had found three potential transcriptional starts sites 5′ to *kpsF* ^20,24^, but to date, only the upstream start (PR1-1) has been studied in detail. We used a 5′ RACE (rapid amplification of cDNA ends) on mRNA extracted from strain UTI89 to confirm that these three start sites exist at +1, +132 and +181 (see Supplementary Figs S1, S2 and Table S1). To quantify expression from the three start points, denoted PR1-1, PR1-2 and PR1-3, we performed quantitative real-time PCR (qRT-PCR) using the primer sets indicated in Fig. 2A on RNA extracted from UTI89 at various time points during growth in LB at 37 °C. The total transcript exiting the PR1 region (detected using primers *kpsF*-F and *kpsF*-R) was the most abundant mRNA (Fig. 2B). Expression peaked in early to mid exponential phase before decreasing through late exponential phase with much lower levels of transcript upon entry into stationary phase (Fig. 2B). This is the first evidence of growth phase dependent transcription from PR1.

Transcription originating from PR1-1 (detected using the primer set 1-1-F and 1-1-R) appears to contribute the least to the total mRNA exiting PR1 and this is true for all stages of growth tested (Fig. 2B). During mid-exponential phase the combined transcript originating from both PR1-1 and PR1-2 (detected using primer set 1-2-F and 1-2-R) is greater than that originating merely from PR1-1 (*p* = 0.039) indicating that PR1-2 is functional on the chromosome of UTI89. However, the transcript from PR1-1 and PR1-2 contributes less than a quarter of the total transcript exiting PR1, indicating that transcription from PR1-3 is accounting for the majority of transcription that exits the PR1 region during exponential growth. In contrast, when the cells enter stationary phase both PR1-3 and PR1-1 appear less active, with the low levels of transcription at this point due to transcription from PR1-2 (Fig. 2B).

Taken together these results clearly show that significant transcription is initiating from all three promoters on the chromosome of UTI89 and that transcription from PR1 is growth phase dependent with the three promoters contributing differently to the total transcript during the growth phase.

To confirm whether these growth phase dependent changes in the level of transcription observed from the PR1 promoter region were mirrored in the level of capsule expression, flow cytometry analysis (FACS), using anti-K1 antibody, of UTI89 at various stages of growth was carried out (Fig. 2C). There is a clear shift in fluorescence intensity from low to high as the cells grow (and hence produce capsule). This fluorescence intensity peaks at around OD_600_ = 1.0 and then appears to fall back which is in line with the decrease in transcription from the PR1 promoter region at late-exponential phase (Fig. 2B). This is the first evidence for the effect of growth phase on the expression of group 2 capsules.

### Transcription initiation at promoter PR1-2 is dependent on the activation of the upstream promoter PR1-1

**Figure 3:**
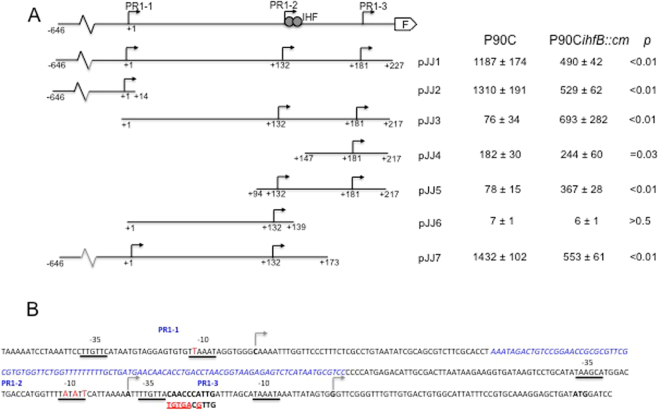
The PR1 region of the *kps* gene cluster. (**A**) The PR1 region has been expanded to show three tandem promoters PR1-1, PR1-2 and PR1-3 and the start of the *kpsF* gene. Filled circles denote an IHF binding site. Various minimal constructs are shown which were cloned upstream of *lacZ* in plasmid pRS415, transformed into P90C or P90C*ihfB::cm* and the corresponding β-galactosidase activity (Miller Units) are shown with *p* values. Values represent the mean of three independent experiments ± the standard error of the mean. (**B**) The nucleotide sequence of the PR1 region. The three promoters are shown and the arrows and bold nucleotide the initiation site for each promoter. The -35 and -10 regions are underlined. The inserted Rho-independent terminator is shown in blue and italics. The mutations in the -10 of PR1-1 and PR1-2 are shown in red in a larger font. The IHF binding site is in bold with the mutated IHF site in UTI189_IHF_ shown in red below the sequence.

To understand in more detail the interaction between the three promoters, a variety of *lacZ* transcriptional fusions were generated (using promoter-probe plasmid pRS415), as shown in Fig. 3A. Plasmid constructs were transformed into strain P90C and β-galactosidase activity was measured for each strain (Fig. 3A). Strains P90C(pJJ1) and P90C(pJJ2), in which the entire PR1 region or just PR1-1 alone was cloned upstream of *lacZ*, demonstrated significant β-galactosidase activity (1187 ± 174 and 1310 ± 191 Miller Units respectively). Likewise strain P90C(pJJ7) that contains PR1-1 and PR1-2 expressed comparable high level β-galactosidase activity (1432 ± 102 Miller Units). This indicates that when the PR1 region is cloned on a multi-copy plasmid PR1-1 is a powerful promoter (Fig. 3A). This in contrast to the qRT-PCR data using mRNA from strain UTI89 (Fig. 2A). Our interpretation of this is that when the PR1 region is on the chromosome in the correct context, then transcription from PR1-1 is repressed somewhat and this regulation is lost on multi-copy plasmid. At this stage we cannot be sure of the mechanism, it may indicate additional regulatory *cis*–acting sequences beyond −646 and/or the supercoiling state or nucleoid organisation. The β-galactosidase activity from P90C(pJJ3) and P90C(pJJ5) was significantly lower (10 fold) than that of the full length PR1 construct (pJJ1) despite these constructs both containing the PR1-2 and PR1-3 transcriptional start sites (Fig. 3A). Furthermore strain P90C(pJJ4) (containing only PR1-3) gave significantly higher β-galactosidase activity than either P90C(pJJ3) or P90C(pJJ5) (Fig. 3A). Plasmid pJJ4 has lost the putative IHF consensus-binding site between +132 and +141, suggesting that IHF may be playing a role in repressing transcription from PR1-3 (see below). Analysis of the β-galactosidase activity from P90C(pJJ6) that only contains PR1-2 showed very low level activity (Fig. 3A). This very low level of transcription from PR1-2 may also explain why plasmids pJJ3 and pJJ5 gave low β-galactosidase activities. These data suggest that when promoter PR1-2 is cloned in isolation it appears to have low transcriptional activity whereas in its context on the *E*. *coli* chromosome it does contribute significantly to the overall transcript exiting the PR1 region. To establish the contribution of PR1-2 to transcriptional activity in pJJ7, that contains PR1-1 and PR1-2 (Fig. 3A), the predicted -10 hexamer of PR1-2 was subject to site directed mutagenesis (Fig. 3B) (see Supplementary Table S2). This mutation in pJJ7 denoted pJJ7_PR2-10*_ showed a 73% reduction in β-galactosidase activity and a 76% reduction in mRNA copy number (Table 1). This is consistent with loss of PR1-2 activity and indicates that PR1-2 is a powerful functional promoter when cloned in the context of PR1-1. One possible explanation is that PR1-2 is dependent on the upstream promoter PR1-1 for activity. To test this hypothesis the predicted -10 hexamer of PR1-1 was subject to site directed mutagenesis with a single base, T → C, substitution (Fig. 3B) (see Supplementary Table S2). This mutation in plasmid pJJ2 that only contains PR1-1 (denoted pJJ2_PR1-10*_) decreased β-galactosidase activity by approximately 99% compared to strain P90C(pJJ2) (Table 1) confirming the mutation had effectively destroyed the PR1-1 promoter. When the same mutation was introduced into plasmid pJJ7, that contains PR1-1 and PR1-2, β-galactosidase activity again reduced by 99% suggesting this mutation was abolishing transcription from both PR1-1 and PR1-2 concurrently. This result was confirmed by qRT-PCR analysis of the constructs with and without the -10 mutation using primers within the *lacZ* gene (Table 1). Overall these data demonstrate that PR1-2 is a functional promoter whose activity is dependent on the upstream PR1-1 promoter.

**Table 1:** Transcriptional activity of mutated PR1-1 and PR1-2 promoters.

Construct in P90C	β-galactosidase activity (Miller Units)	mRNA copy no./μg RNA
pJJ2	3640 ± 564	3.13 × 10^8^ ± 2.5 × 10^6^
pJJ2_PR1-10*_	54 ± 10	7.01 × 10^6^ ± 1.23 × 10^5^
pJJ7	1505 ± 141	1.04 × 10^8^ ± 8.24 × 10^6^
pJJ7_PR1-10*_	16 ± 9	5.46 × 10^6^ ± 3.65 × 10^5^
pJJ7 _PR2-10*_	400 ± 53	2.5 × 10^7^ ± 2.23 × 10^6^

**Figure 4:**
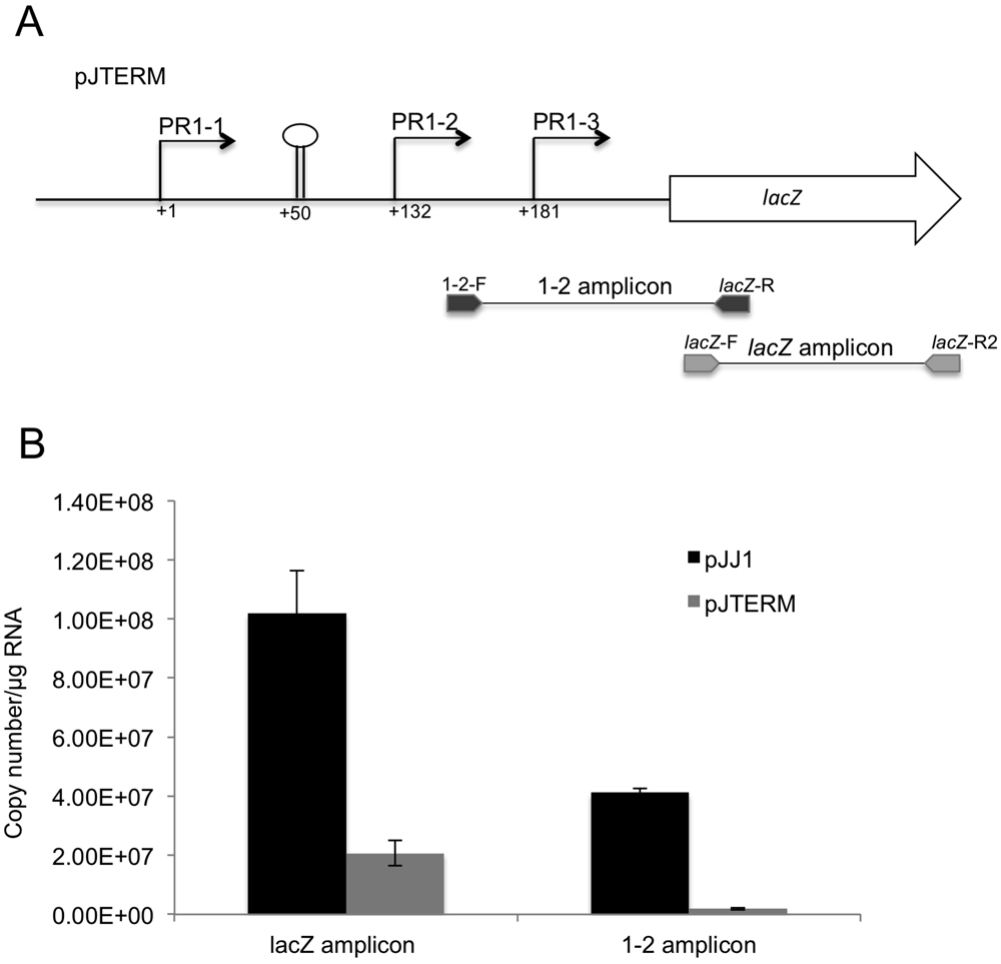
Transcription initiation from PR1-2 is dependent on transcription from PR1-1. (**A**) A Rho - independent transcriptional terminator (depicted as a stem-loop structure) was introduced at the position indicated and the primer sets shown were used to measure transcription initiating from PR1-2 and PR1-2/PR1-3 combined. (**B**) P90C containing plasmids pJJ1 or PJTERM was grown to mid-exponential phase (OD_600_ between 0.5-0.6) RNA was extracted and qRT-PCR was carried out using primer sets shown in (**A**). The results represent the mean of 3 individual experiments (normalised against *rpoD* and 16 s transcripts) carried out in quadruplicate. Error bars represent the standard error of the mean.

To understand how the promoter activity at PR1-1 affects the activity of PR1-2, plasmid pJTERM was constructed (Table 2) in which a Rho-independent transcription terminator based on the *E*. *coli mraY* terminator^25^ (see Supplementary Table S3) was inserted at position +50 in plasmid pJJ1 (Fig. 3B). To test the effect of the terminator on transcription a β-galactosidase assay was carried out on P90C(pJJ1) and P90C(pJTERM). As previous P90C(pJJ1) gave 1023 ± 71.5 Miller Units while P90C(pJTERM) gave 140 ± 17.1 Miller units which is consistent with loss of both PR1-1 and PR1-2 activity with only PR1-3 initiating transcription of the *lacZ* gene (compare Miller units for pJJ4 Fig. 3A). To further confirm this result qRT-PCR was carried out on P90C(pJJ1) and P90C(pJTERM) using the primers indicated in Fig. 4A. The results (Fig. 4B) show that insertion of the terminator stopped transcription from PR1-2 with a 95.6% reduction in mRNA copy number from pJTERM compared to pJJ1 using the primers 1–2 F and *lacZR*1 (Fig. 4A). With the *lacZ* primers (measuring the total transcript exiting the promoter region) we see a 79.1% reduction in copy number from pJTERM compared to pJJ1, which is consistent with transcription still initiating from PR1-3. These data confirm the dependence of PR1-2 on transcription coming from PR1-1 and that transcription from PR1-3 is independent of PR1-1. One mechanism by which transcription from PR1-1 could activate PR1-2 is by a transcription-coupled DNA supercoiling (TCDS)^26^. In this scenario RNAP creates local supercoiling during transcription with negative supercoils generated upstream and positive supercoils downstream of the translocating RNA^26^. In this case PR1-2 would be a supercoiling sensitive promoter such that TCDS from PR1-1 and PR1-3 might be the source of the local DNA supercoiling for promoter activation. To establish if PR1-2 is supercoiling sensitive, strain P90C(pJJ6) was grown in the presence of 5 μgml^−1^ of novobiocin, an inhibitor of the GyrB subunit of the DNA Gyrase enzyme^27^ and β-galactosidase activity measured. The presence of novobioicin caused an increase in β-galactosidase activity from 8 ± 1.3 Miller units to 19 ± 3 Miller units at mid log phase (*p* = 0.001) indicating that PR1-2 is sensitive to supercoiling. Both PR1-1 and PR1-3 were unaffected by the addition of novobiocin, indicating that these two promoters are not supercoiling sensitive (data not shown).

### Growth phase dependent regulation of transcription by IHF

There is an IHF consensus binding site in the UTR centred at +140 that has been shown to bind IHF^20,28^. In these previous studies only transcription initiating at PR1-1 was considered. To address the role of the IHF binding site at +140 in regulating transcription from PR1-2 and PR1-3, a number of the promoter-*lacZ* constructs were introduced into strain P90C*ihfB::cm* and the level of β-galactosidase activity assayed (Fig. 3A). In strains harbouring pJJ2, which contains just the PR1-1 promoter with no UTR or any IHF binding sites the *ihfB* mutation significantly reduced β-galactosidase activity (Fig. 3A). This confirms the earlier findings that IHF plays an indirect role in activating transcription from PR1-1^28^. In contrast, P90C*ihfB::cm* carrying plasmid pJJ5 that extends from +94 in the UTR and contains both the IHF binding site and PR1-2 and PR1-3 showed an increase in β-galactosidase activity (Fig. 3A). However there was no difference in β-galactosidase activity between strains P90C and P90C*ihfB::cm* carrying pJJ4, that lacks the IHF site at +140 (Fig. 3A). These data together with the observation that P90C(pJJ5) had lower β-galactosidase activity than P90C(pJJ4) (Fig. 3A) indicates that IHF binding at +140 acts to repress transcription from PR1-2 and PR1-3. To confirm that we were not seeing effects due to IhfA homodimer formation as reported previously in other systems^29^ we assayed the β-galactosidase activity of strains P90C*ihfA::Tn10*(pJJ1) and P90C*ihfA::Tn10*(pJJ5) lacking the IhfA subunit. As predicted the *ihfA* mutation decreased β-galactosidase activity from 1240 ± 110 Miller units to 947 ± 93 Miller units (*p* = 0.0176) in the case of pJJ1 and increased β-galactosidase activity from 54 ± 6 Miller units to 349 ± 29 Miller Units (*p* = 0.00006) in the case of pJJ5. As such we can be sure that heterodimeric IHF is acting at the +140 site.

**Figure 5:**
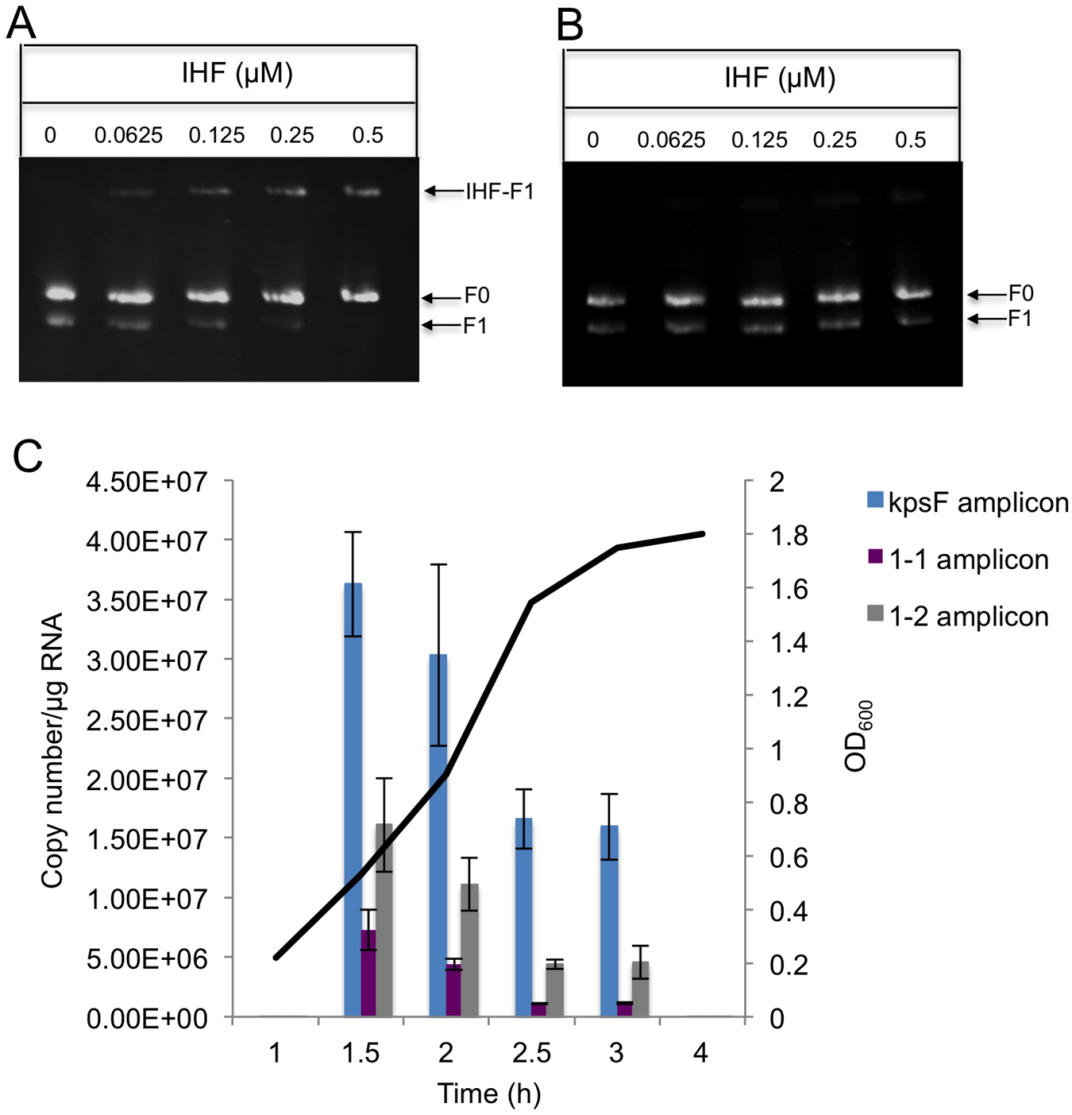
Mutation of the cis-acting IHF binding site at +140 in the PR1 region dramatically changes the growth phase pattern of transcription. The IHF binding site at +140 at the capsule locus on the chromosome of UTI89 was mutagenized by SDM (strain UTI89_IHF_). EMSAs were carried out on fragments (+46 to +224) amplified from the UTI89 (**A**) and UTI89_IHF_ (**B**) chromosome using increasing concentrations of purified IHF. F1 represents the test fragment while F0 represents the free negative control DNA. An IHF-DNA complex is clearly seen with the wild-type fragment (indicated by an arrow in the Panel A) at 0.0625 μM IHF while the mutant fragment only shows partial binding at the highest concentrations of IHF. (**C**) UTI89_IHF_ was grown at 37 °C and at the time points indicated samples were taken OD_600_ measured and RNA extracted for qRT-PCR analysis using the primer sets shown in Fig. 2A. The results represent the mean of three independent experiments (normalised against *rpoD* and 16 S transcripts). Error bars represent the standard error of the mean. The growth curve is a representative of one independent experiment but OD_600_ values were within 0.05 units for each sample. The images from two separate gels were not manipulated but cropped for clarity. The original un-cropped images are in the Supplementary material (Fig. S4).

To specifically dissect out the *cis*-activity of IHF in regulating transcription at PR1, an IHF binding site mutant was constructed in strain UTI89 (UTI89_IHF_) as described in experimental procedures. In this strain the consensus IHF binding sequence 5′-TTACAACCCATTG^30^ was replaced by 5′-TTATGTGACGTTG – the mutated nucleotides are underlined (Fig. 3B). The first 3 nucleotides of the consensus sequence were not mutated as these form the last part of the predicted −35 sequence for PR1-3 (Fig. 3B). To establish that the mutated IHF site no longer bound IHF, two PCR fragments (F1 and F2) spanning from +46 to +224 were amplified using primers EMSA-IHF-F (5′-GCACCTCCATGAGACATT-3′) and EMSA-IHF-R (5′-CAGCTCCTTTGCACGG-3′) from UTI89 and UTI89_IHF_ respectively. These fragments were then subjected to EMSA using increasing concentrations of purified IHF (Fig. 5). The F1 fragment begins to shift at 0.0625 µM IHF with all of the DNA binding at the highest concentration of IHF (0.5 µM) (Fig. 5A) whereas only a very faint band shift was detected with mutated F2 fragment at the highest concentration of IHF (Fig. 5B) confirming the binding of IHF to the mutated site is largely diminished.

To determine the effect of mutating the IHF binding site on transcription qRT-PCR was carried out on strain UTI89_IHF_ using the primer sets described previously (Fig. 1A). The results are shown in Fig. 5C. The first striking observation was that mutating the IHF binding site abolished the growth phase dependent transcription with no significant difference (*p* > 0.05) between the total transcript exiting the PR1 region (detected using primers *kpsF*-F and *kpsF*-R) at any of the time points (Fig. 5C). Any peak in transcription occurred much earlier in exponential phase (Fig. 5C). Critically the contribution of PR1-3 to the total transcript coming from the PR1 promoter region at the end of exponential and the start of stationary phase is clearly different between UTI89 and UTI89_IHF_ (Compare Figs 2B and 5C). In UTI89_IHF_ in stationary phase the bulk of transcription exiting PR1 is from PR1-3 which, is in keeping with IHF binding at the IHF site at +140 and repressing transcription from this promoter as the cells reach the end of exponential phase and enter stationary phase. This confirms that the IHF site at +140 acts to repress transcription from PR1-3 upon entry to stationary phase and reduce the levels of capsule expression. To confirm a role for IHF in the growth phase regulation from PRI the relative amounts of transcript from each promoter was determined by qRT-PCR in strain UTI89*ihfB*::*cm* lacking IHF. As predicted the *ihfB* mutation results in a 6.6 fold reduction in the total mRNA copy number exiting from PR1 promoter region (see Supplementary Fig. S3) confirming the indirect role of IHF in activating transcription from PR1^28^. However, what was striking was that in the *ihfB* mutant the level transcript exiting the promoter region (*kpsF* amplicon) does not decrease as the cells move into stationary phase (see Supplementary Fig. S3), which is unlike the temporal pattern observed for the wild type strain (Fig. 2B). These data confirm that IHF plays a role in the growth phase dependent expression from PR1.

## Discussion

In this study we have demonstrated the presence of three functional tandem promoters PR1-1, 1-2 and 1-3 in the PR1 promoter region of the *E*. *coli kps* capsule gene cluster. In *E*. *coli* it has been estimated that 25% of the transcription units are have more than one promoter and it has been proposed that multiple promoters may also serve as back up promoters ensuring that potentially deleterious mutations that abolish the function of one promoter do not lead to the loss of expression of a factor essential for growth and survival^31^. In addition, multiple promoters may be regulated independently by the activity of different activator or repressors and thereby may allow integration of different environmental stimuli to differentially regulate transcription of a particular gene^32–35^. As such, multiple promoters at PR1 may permit the integration of different signals and regulators at this site potentially allowing adaptation to new environments that may be encountered either in the transition into or out of the host or at different sites within the host. One possibility is that following temperature upshift from 20 °C to 37 °C and the switching on of transcription from PR1 and capsule expression^23^ the three promoters are used differentially to provide an initial burst of transcription to rapidly encapsulate the bacterium. However, we found no difference in the patterns of expression from the promoters in PR1 following temperature upshift (Jia unpublished results). As such at this stage we cannot preclude that these promoters may function to differentially control capsule expression in different niches within the host. The observation that PR1-1 had significantly more activity when cloned independently on a multi-copy plasmid suggests that additional regulation is occurring when the PR1-1 promoter is located on the chromosome and in single copy. One possibility is that on multi-copy PR1-1 is titrating out a repressor and we are currently investigating this.

The three promoters PR1-1, PR1-2 and PR1-3 contribute differentially to the total transcript that exits the PR1 promoter region. By both site directed mutagenesis to disrupt the -10 of PR1-1 and by introduction a Rho independent terminator between the two promoters we demonstrate that transcription initiation from PR1-2 is dependent on transcription initiation from PR1-1. Our interpretation of this is that transcription from PR1-1 could activate PR1-2 by TCDS^26^, in which RNAP creates local supercoiling during transcription with negative supercoils generated upstream and positive supercoils downstream of the translocating RNA^36^. In this scenario, PR1-2 would be a supercoiling sensitive promoter such that transcription-induced DNA positive supercoiling from PR1-1 could activate its expression. The observation that novobiocin, which inhibits GyrB and increases positive supercoiling, leads to increased transcription from PR1-2 indicates that PR1-2 is a supercoiling sensitive promoter activated by increased positive supercoils. This is in keeping with our hypothesis of TCDS in activating expression from PR1-2. Previous studies have demonstrated that promoter-promoter interaction via TCDS is normally short-range^37^, the separation between PR1-1 and PR1-2 is around 132 bp, so the notion that transcription from PR1-1 can influence the activity of adjacent promoter PR1-2 on the same DNA chain though local supercoiling is therefore reasonable. In the *fis* promoter there are three tandem RNAP binding sites and it has been proposed that idling of RNAP at an upstream promoter acts as an accessory factor to stimulate transcription from the downstream promoter^38^. The observation that the mutation at position 2 of the -10 of PR1-1, prevented transcription from PR1-2, would argue against RNAP idling at PR1-1 being the mechanism by which PR1-2 is activated, since mutations at this site in the -10 region will not prevent RNAP binding and idling at the PR1-1 promoter^39^.

Critically we show that transcription from the PR1 promoter region is growth phase dependent with the contribution of each promoter differing during the growth phase. The qRT-PCR data shows that transcription peaks early in mid-exponential phase but rapidly declines by late exponential phase and entry into stationary phase (Fig. 2B). In mid-exponential phase PR1-1 and PR1-2 appear to contribute less than a quarter of the total transcript while PR1-3 appears to contribute the most (Fig. 2B). In contrast by entry into stationary phase transcription from PR1-3 has diminished with transcription from PR1-1/1-2 accounting for the low level of transcription (Fig. 2B). The FACS data confirmed that the observed pattern of transcription is mirrored in reduced capsule expression in entering stationary phase. This is the first evidence for growth phase regulation of transcription of the *kps* gene cluster and group 2 capsule expression in pathogenic *E*. *coli*. Implicit in this is that PR3 is also growth phase dependent since transcription from PR3 will control expression of region 2 and expression of the polysaccharide biosynthesis genes (Fig. 1). This reduction in capsule expression on approaching stationary phase may reflect a fine balance in terms of the level capsule expression required to facilitate stationary phase survival^2,15^ versus the loss of energy in terms of exporting carbohydrate out of the cell.

Previously it had been hypothesized that IHF played a dual role in activating transcription from PR1-1^28^, acting indirectly through regulation of other as yet unidentified regulators of PR1-1 and directly through binding at the IHF site at +140^28^. In this paper we establish by mutagenesis of the IHF site at +140 a clear direct role for IHF binding at this site in inhibiting transcription from PR1-2 and PR1-3 rather than activating PR1-1 and demonstrate that this binding is critical in the growth phase regulation of transcription. The apparent discrepancy to previous studies can be explained by the fact that in previous studies the IHF site was deleted rather than disrupted by site directed mutagenesis^28^. Based on data in this paper we now know that this deletion would have also deleted the -35 of PR1-3 thereby abolishing this as a functional promoter. As such, this deletion would have reduced the total transcription from PR1 and thereby lead to the incorrect conclusion that binding of IHF at +140 was required for activation of transcription from PR1-1. The phenotype of *ihf* mutants, namely an overall decrease in the level of transcription coming from PR1 together with a loss of growth phase dependent regulation would be in keeping for this dual role of IHF acting indirectly to stimulate transcription of PR1-1 and acting directly to repress transcription from PR1-2 and PR1-3 upon entry into stationary phase. IHF is believed to regulate 150 genes in *E*. *coli* at the level of transcription of which two thirds are activated by IHF^40^. The levels of IHF increase in early stationary phase^41,42^ and it has been shown to regulate gene expression during the physiological changes associated with the transition from exponential to stationary phase^43^. In our model we propose that as the levels of IHF rise approaching the end of exponential phase it binds at +140, which overlaps the -35 hexamer region of PR1-3. This binding would cause a U-turn as the DNA wrapped around the protein^30^, which may prevent the RNAP recognizing the promoter element of PR1-3 efficiently, or render the DNA to conform in a way that precluded the stable binding of RNAP or later steps of the initiation cycle^39^. Likewise binding of IHF here could create a roadblock to block transcription initiating from PR1-2^44^. Of course we know that regulation of transcription from PR1 is complex involving in addition H-NS and SlyA as well as a yet unidentified IHF regulated activator of transcription^20,23^. As such the regulation of transcription at PR1 will involve a complex nucleoprotein structure at this site containing a number of regulatory proteins with multiple protein:protein and protein:DNA interactions. The data in this paper demonstrate that any models of regulation of PR1 must include the additional complexity of three tandem promoters within this region.

## Methods

### Bacterial Strains and Plasmids

The strains and plasmids used in this study are listed in Table 2. Bacteria were routinely grown in Luria-Bertani (LB) medium at 37 °C, and supplemented with antibiotics as appropriate at the following concentration: 50 μg/ml^−^ kanamycin, 100 μg/ml ampicillin and 25 μg/ml chloramphenicol. Strain UTI89*ihfB*::*cm* was constructed by P1*vir* transduction of recipient strain UTI89 by donor strain P90C*ihfB*::*cm* as described previously^45^.

**Table 2:** Bacterial strains and plasmids used in this study.

Strain	Genotype	References/Source
DH5α	*F-*, *Φ80lacZΔM15*, *Δ*(*lacZYA-argF*), *U169*, *recA1*, *endA1*, *hsdR17* (*rK–*, *mK*+), *phoA*, *supE44 λ– thi-1 gyrA96*, *relA1*	^50^
P90C	*F-*, *ara*, *Δ*(*lacZ-pro*), *thi-1*	^51^
P90C*ihfA::Tn10*	P90C x P1*vir* (DS940*ihfA::Tn10*)	This study
P90C*ihfB::cm*	P90C x P1*vir* (MS105 *ihfB::cm*)	This study
UTI89	K1+, cystitis-derived isolate of serotype O18:K1:H7	^52^
UTI89*ihfB::cm*	UTI89 x PI*vir* (P90C *infB::cm*)	This study
UTI89_IHF_	UTI89 with IHF binding site at capsule locus mutated from TTACAACCCATTG to TTATGTGACGTTG	This study
**Plasmid**	**Relevant Characteristics or Features**	**Reference/Source**
pRS415	Transcriptional fusion reporter plasmid, *lacZ* promter-probe vector	^51^
pJJ1	Region -646 to +227 of PR1 cloned upstream of lacZ in pRS415	This study
pJJ1_PR1–10*_	Single site substitution (T → C) at -13 of PR1–1 in pJJ1.	This study
pJJ2	Region -646 to +14 of PR1 cloned upstream of *lacZ* in pRS415	This study
pJJ2_PR1–10*_	Single site substitution (T → C) at -13 of PR1-1 in pJJ2	This study
pJJ3	Region +1to +217 of PR1 cloned upstream of *lacZ* in pRS415	This study
pJJ4	Region +147 to +217 cloned upstream of *lacZ* in pRS415	This study
pJJ5	Region +94 to +217 cloned upstream of *lacZ* in pRS415	This study
pJJ6	Region +1 to +139 cloned upstream of *lacZ* in pRS415	This study
pJJ7	Region -646 to +173 cloned upstream of *lacZ* in plasmid pRS415	This study
pJJ7_PR1-1-10*_	Single site substitution (T → C) at -13 of PR1-1 in pJJ7	This study
pJJ7_PR1-2-10*_	Triple site substitution TATATT → TCTCTC at -10 hexamer of PR1-2	This study
pJTERM	Rho independent terminator inserted at +50 of PR1 in pJJ1	This study
pDOC-C	Gene doctoring vector	^47^
pACBSceI	1 Sce1, λ-Red vector	^47^

### β-Galactosidase Assays

Assays were carried out essentially as described^46^. Triplicate overnight cultures were diluted 1:100 into fresh, pre-warmed LB broth supplemented with appropriate antibiotics and grown to mid-exponential phase. 100 µl aliquots of each mid-log culture were mixed in with 900 µl Z-Buffer (0.85% w/v Na_2_HP0_4_, 0.55% w/v NaH_2_PO_4_, 0.07% w/v KCl, 0.025% w/v MgSO_4_) to which 2.7 µl of 2-Mercaptoethanol (0.05 M) was added and the cell lysed by the addition of 40 µl choloroform, 20 µl 0.01% (w/v) SDS. After lysis 35 µl O-nitrophenyl-β-D-galactoside (ONPG: 4 mg/ml in Z-Buffer) was added to 176 µl of sample and the reaction proceeded in the dark at 28 °C until a yellow color developed when the reaction was terminated by the addition of 88 µl 1 M Na_2_CO_3_ to all wells. The plates were then read by measuring the OD_420nm_ in plate reader (Synergy™ HT Multi-Mode Microplate Reader) for each reaction and the β-galactosidase activity in Miller units determined using the equation:

$${\rm{\beta }}-{\rm{galactosidase}}\,{\rm{activity}}={{\rm{OD}}}_{420{\rm{nm}}({\rm{test}})}-{{\rm{OD}}}_{420({\rm{blank}})}/{{\rm{OD}}}_{600}{\rm{\times }}{\rm{T}}{\rm{\times }}{\rm{V}}$$

where T = time (min), V = volume (ml), and 1 Miller Unit is equivalent to the amount of enzyme which produced 1 µmol O-nitrophenol/min.

### Construction of UTI89 ihf binding site mutant

The gene doctoring method^47^ was used to construct strain UTI89_IHF_. A 801 bp fragment containing the IHF binding site was amplified by PCR from UTI89 (using primers kpsF-F and kpsF-R) and was cloned in pBluescript SK+. The IHF binding site mutated by site directed mutagenesis (described below) and the mutated fragment was then subcloned into pDOC-C downstream of a Kan cassette. A 588 bp upstream fragment amplified by PCR from the UTI89 chromosome (using primers Fup-F and Fup-R) was then cloned upstream of the Kan cassette to provide the left flank for homologous recombination. The resulting plasmid was transformed into UTI89 alongside pACBSce1. Transformants were patched on 5% sucrose before induction of recombination onto the chromosome as described previously^47^. The introduction of the IHF binding site mutation on the chromosome of UTI89 was confirmed by PCR and sequencing.

### RNA extraction

For RNA extraction, overnight *E*. *coli* cultures were inoculated into fresh LB medium (1:100) and grown at 37 °C to the appropriate OD_600_. 1 ml of culture was extracted and immediately mixed with 2 ml RNA Protect Bacteria Reagent (Qiagen). RNA was subsequently extracted using an RNAEasy Mini Kit according to the manufacturers instructions (Qiagen). RNA was quantified using a NanoDrop ND-1000 spectrophotometer (NanoDrop), where an *A*_260_ of 1.0 equals 40 μg ml^−1^.

### Quantitative Real-Time PCR (qRT-PCR)

Reverse transcription of RNA was performed using QuantiTect Reverse Transcription Kit for fast cDNA synthesis enabling sensitive real-time two step RT-PCR for gene expression analysis (Qiagen) with 1 µg total RNA. Quantitative real-time polymerase chain reaction (qRT-PCR) was performed on an ABI Prism sequence detector (Applied Biosystems) using FAST SYBR Green Master Mix (Applied Biosystems). The thermal cycling conditions were as follows: 20 s holding step at 95 °C was followed by 40 cycles of a 1 s denaturation step at 95 °C and a 30 s annealing/polymerisation step at 60 °C. For each primer set a standard curve was obtained for absolute quantification by plotting the threshold cycle against the logarithm of a known amount of copy numbers and the quantities of target copies contained in an unknown sample were determined by extrapolation from the linear regression of the standard curve. A negative control (just RNA) that had not undergone the reverse transcription step was also included as a negative control in each run. In addition, each set of primers were also checked every time to make sure no primer-dimers occurred and that there was no other DNA contamination in the reaction by carrying out a melting curve stage after the total amplification cycle (rapid heating up to 95 °C for 15 s to denature the DNA, followed by cooling to 60 °C for 1 min and increasing 0.3 °C s^−1^ to 95 °C for 15 s).

### Site Direct Mutagenesis by PCR

This protocol was performed as described previously^48^. A pair of complemented mutagenic primers was designed containing the desired substitution of nucleotides flanked by ~20 bp unmodified nucleotides on each side of the mutation site. Each 50 µl PCR reaction contains 5 µl 10 × *Pfu* Ultra buffer (Stratagene), 2 µl of 25 ng µl^−1^ template plasmid DNA, 12.5 µl of 10 ng µl^−1^ of each primer, 2.5 µl of 2.5 mM dNTP, 1 µl Strategy Pfu Ultra polymerase (Stratagene) and 14.5 µl ddH_2_0. PCR was carried out under the following cycles: 95 °C × 5 min (1 cycle) 95 °C × 50 sec, 60 °C × 50 sec, 68 °C × 1 min + 1 min per 1 kb template (30 cycles), 7 min × 68 °C (1 cycle). The PCR product was immediately digested with Dpn I before transforming into *E*. *coli* DH5α and then plated out on the LB plate with appropriate antibiotics. Successful mutated plasmids was purified from cultured single colony and verified by sequencing.

### Protein purification

Briefly, IHF was overexpressed in *E*. *coli* strain K5746 containing plasmid pP_L_hiphimA-5 and purified using a heparin column as described^49^.

### Electrophoretic Mobility Shift Assay (EMSA)

Competitor DNA and the promoter regions fragment were amplified from pBluescript or the promoter constructs with primers designed to anneal to the plasmid backbone either side of the cloning site. For each construct, PCR amplification products were checked by agarose gel electrophoresis and purified by MiniElute PCR purification kit (Qiagen). Equal quantities (25ng) of the target and competitor DNA were mixed and added to binding reactions containing reaction buffer (5 × Reaction binding buffer: 10 nM TrisCl pH 7.8; 0.5 mM DTT; 1.25% (v/v) glycerol; 25 µM spermidine) and varying concentrations of purified IHF which had been pre-equilibrated for 10 min. Polyacrylamide gels underwent preliminary electrophoresis in 1 × TBE at 90 V for 90 min before samples loading. Samples were run into the gel at 250 V for 3 min before being separated at 120 V for 60 min. One lane containing DNA loading buffer was used as an indicator for migration of DNA fragments through the gel. Following electrophoretic fractionation of samples, gels were stained with 0.5 µg/ml Ethidium Bromide for 30 min and visualized under UV light.

### Flow Cytometry Analysis

Bacteria were grown to the OD indicated. Around 10^8^ cells were washed three times in PBS, blocked for 1 h in 1% (w/v) BSA/PBS and treated with anti-K1 Ab (1/400 dilution) in buffer A [1% (w/v) BSA/PBS, 0.05% (v/v) Tween 20] for 1 h at RT. After one wash with buffer A bacteria were treated with secondary Ab (1/500 dilution donkey anti-mouse Alexa Fluor 594) in buffer A for 1 h at RT and then washed three times in buffer A. 100,000 cells were analyzed for fluorescence per sample using a BDFortessa flow cytometer.

### Data availability

All data generated or analysed during this study are included in the published article (and its Supplementary Information files).

### Equipment and Settings

The gel images for Fig. 4 were obtained using a UViTEC trans-illuminator and UViDOC software (UViTEC Pro1). The images were not manipulated but cropped for clarity; the original un-cropped images are in the Supplementary material (Fig. S4).

## Electronic supplementary material

Supplementary data
