# Supplementary material for: Three tandem promoters, together with IHF, regulate growth phase dependent expression of the Escherichia coli kps capsule gene cluster
Source: Sci Rep. 2017 Dec 20;7:17924. doi: 10.1038/s41598-017-17891-0 (PMC5738388; doi:10.1038/s41598-017-17891-0)
Supplement: Supplementary file 1 — Supplementary data [file 41598_2017_17891_MOESM1_ESM.doc]

Three tandem promoters, together with IHF, regulate growth phase dependent expression of the *Escherichia coli* *kps* capsule gene cluster.

Jia Jia^a,b^, Jane E. King^a^, Marie C. Goldrick^a^, Esraa Aldawood^a^ and Ian S. Roberts^a^ #

^a^School of Biological Sciences, Faculty of Biology Medicine and Health, Manchester Academic Health Science Centre, University of Manchester, Manchester M13 9PT, UK.

^b^Current Address: Nanjing Medical University, School, 140 Hanzhong Road, Nanjing 210029, China,

#Address correspondence to Ian S. Roberts, [i.s.roberts@manchester.ac.uk](mailto:i.s.roberts@manchester.ac.uk)

**
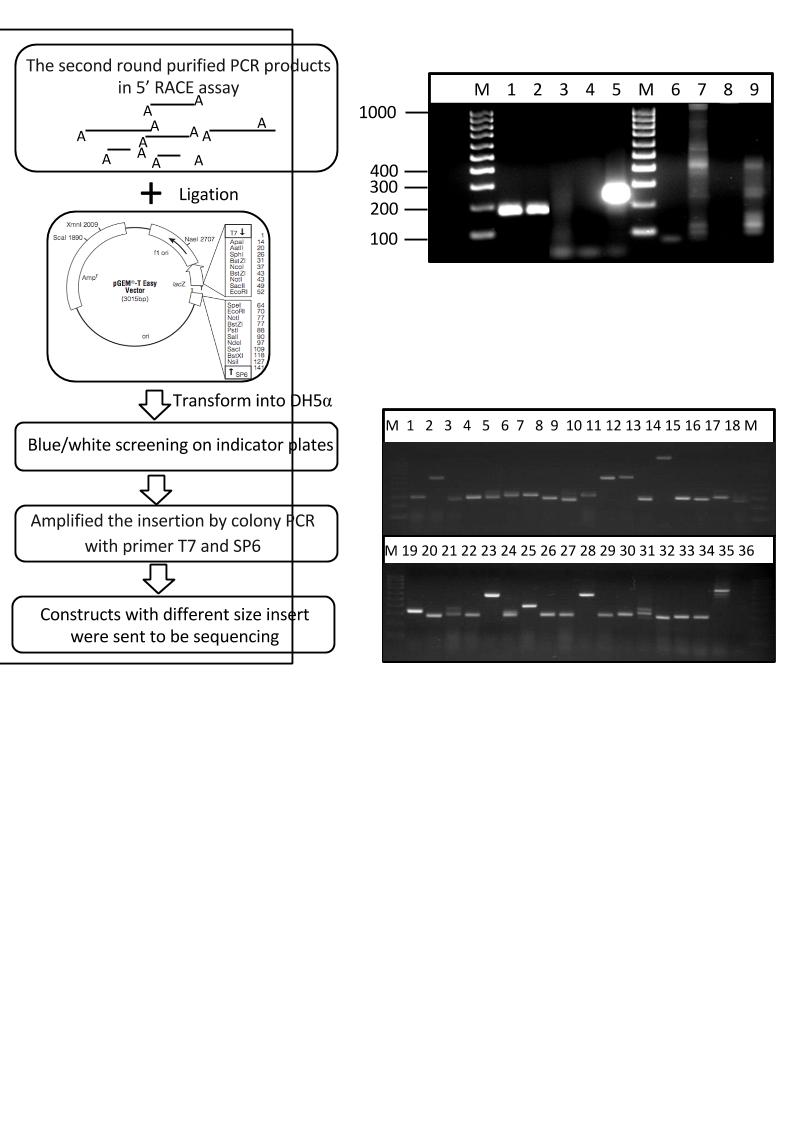
**

**Figure S1. The 5’RACE reaction using mRNA extracted from strain UTI89 grown at 37 ^0^C.** Lane 1 to 2, the control of first-strand cDNA synthesis. The band around 170 bp indicated the cDNA synthesis has been successful. Lane 3, 4, 6 and 8, the negative control showed no DNA contamination of RNA sample and the 5’RACE reactions. Lane 5, the positive control showed the 218 bp 5’UTR region amplified by primers 5’-AGCCTGAATTCCAAAATTTGGTT

CCCTTTCTCG-3’ and 5’-AATCAGGATCCCCTTTGCACGGAAATAATGC-3’ using plasmid pJJ1 as template. Lane 7, the first round PCR products of 5’RACE reaction. Lane 9, the second round PCR products of 5’RACE reaction. M, Hyperladder IV DNA markers (bp), Agarose gel, 2% (w/v)


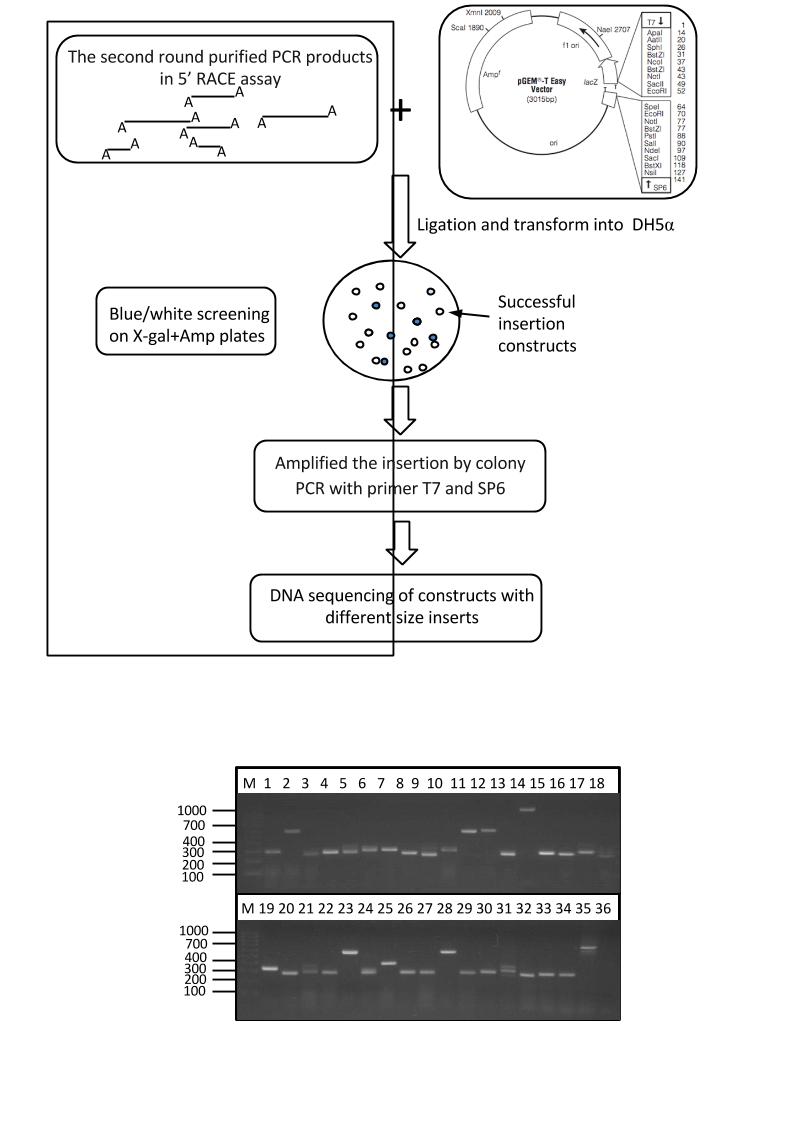


Figure S2 Representative colony PCR using forward and reverse universal sequencing primer of DH5α colonies following ligation of 5’-RACE products from UTI89 into pGEM-T easy. Lanes 1- 35, the colony PCR products. Lane 36, the blank negative control showed no DNA contamination. M, Hyperladder IV DNA markers (bp) are depicted on the side of the agarose gel.

**Figure S3.**


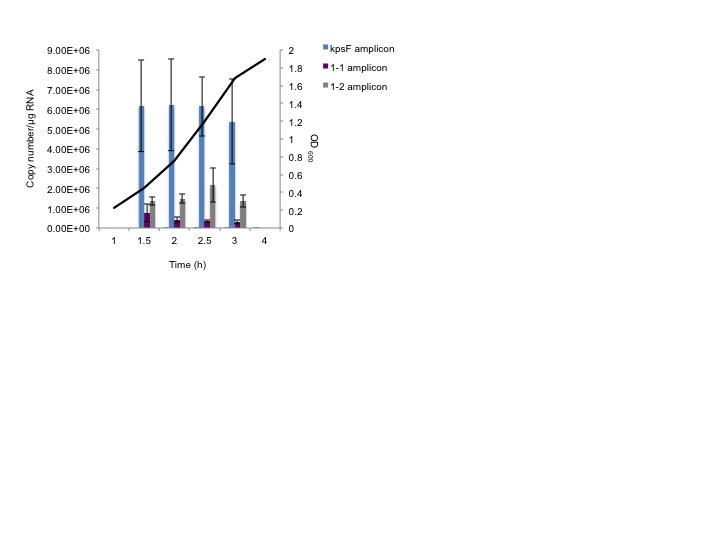


**Fig. S3.** **Reduction and loss of the temporal pattern of transcription in a UTI89*ihf::cm* mutant.** During growth of UTI89*ihf::cm* at 37 °C samples were taken at the time points indicated, OD_600_ measured and RNA extracted for qRTPCR analysis using the primer sets shown in Fig 2A. The results represent the mean of three independent experiments (normalised against *rpoD* and 16S transcripts). Error bars represent the standard error of the mean. The growth curve is a representative of one independent experiment but OD_600_ values were within 0.05 units for each sample.

**Figure S4**


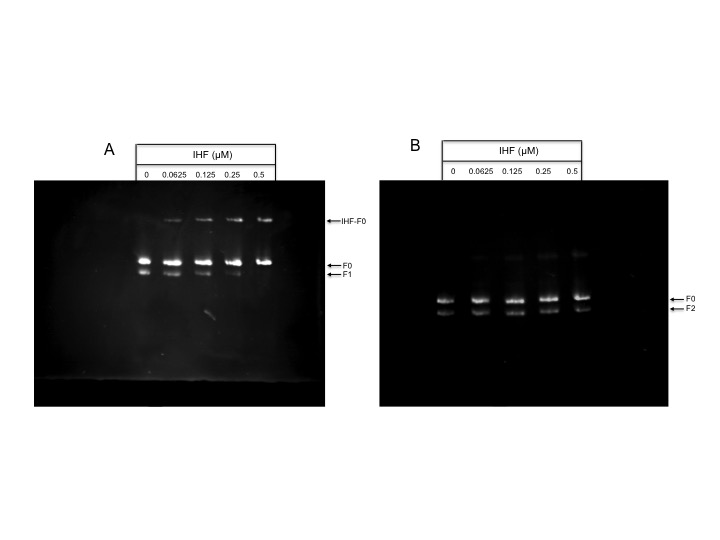
Full length un-cropped gels used in Figure 4.

**
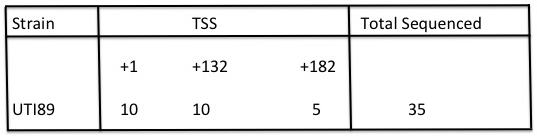
**

**Table S1. Summary of Transcription Start Sites (TSS) from sequenced pGEM-T clones.** The numbers indicate the number of cloned RACE products corresponding to the three TSS (see Figure 3A for numbering). The remaining 10 clones of the 35 sequenced did not contain sequences that corresponded to any sequences in the UTR.

**Table S2 Mutated -10 hexamer sequence of PR1-1 and PR1-2**

| **Promoter** | **Sequence (5’** 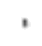**3’)** |
| --- | --- |
| PR1-1 | GAGTGTTAAATAGGTGGGCA^+1^ |
| PR1-1_-10*_ | GAGTGTCAAATAGGTGGGCA^+1^ |
| PR1-2 | TGGTTTTATATTCATTAAAAA^+1^ |
| PR1-2_-10*_ | TGGTTTTCTCTCCATTAAAAA^+1^ |

| Name | A-rich region | Stem-loop | Loop | Poly-T Tail |
| --- | --- | --- | --- | --- |
| *mraY* | tgaaaggctgg | ccggaaccgcgcgtcattgtgcgtttctgg | tcat | attatttcgctgat |
| TERM | aaatagactgt | ccggaaccgcgcgTTCGcgtgTgGttctgg | TTCG | ttTtttTTTgctgat |

**Table S3. Sequence of Rho-independent terminator**

The sequence of the Rho-independent terminator (TERM) inserted at +50 in plasmid pJJ1, the sequence is based on the Rho-dependent terminator 3’ to the *E. coli* *mraY* gene (1) having a 11 nucleotide A-rich region upstream of a stable stem loop structure containing a tetra-loop TTCG and followed by a 9 nucleotide poly-T tail.

1.Lesnik E. A., R. Sampath, H. B. Levene, T. J. Henderson, J. A. McNeil, & D. J. Ecker. Prediction of rho-independent transcriptional terminators in *Escherichia coli.* *Nucleic Acids Res.* **29,** 3583–3594 (2001)
